# A Rare Case of Paxlovid-Induced Pancreatitis

**DOI:** 10.7759/cureus.36528

**Published:** 2023-03-22

**Authors:** Syed Muhammad Hussain Zaidi, Peter A Iskander, Khalid Ahmed, Fouad Jaber, Merlin Paz, Ali Khan, Fahad Malik, Mark M Aloysius

**Affiliations:** 1 Internal Medicine, The Wright Center for Graduate Medical Education, Scranton, USA; 2 Internal Medicine, University of Missouri Kansas City, Kansas City, USA; 3 Internal Medicine, Geisinger Hospitals, Scranton, USA; 4 Internal Medicine, United Health Services, Binghamton, USA

**Keywords:** oncology, protease inhibitor, covid-19, paxlovid, drug induced pancreatitis

## Abstract

Acute pancreatitis can result secondary to an inflammatory cascade due to an insult to the pancreatic parenchyma, whether it be from infections, medications, etc. We present a case of a 37-year-old male with acute pancreatitis after being started on Paxlovid, a combination drug containing Nirmatrelvir and Ritonavir, for COVID-19 treatment. Multiple reports in the literature have documented such an association between acute pancreatitis and the protease inhibitor Ritonavir. We suspect that similar results may have taken place that link the initiation of this medication with pancreatic inflammation.

## Introduction

Pancreatitis can be characterized as an inflammatory cascade to the parenchyma due to various factors, whether it be from infection, medications, trauma, etc. In the United States, the most common cause of acute pancreatitis (AP) is secondary to gallstones. Other causes include alcohol, hypertriglyceridemia, autoimmune disease, or secondary to pancreatic manipulation, such as endoscopic retrograde cholangiopancreatography (ERCP). The annual incidence of AP in the United States is 4.9-35 per 100,000 population and accounts for roughly 200,000 hospital admissions per year: drug-induced pancreatitis accounts for only <5% of the cases [1]. Microorganisms, including viruses, have been reported to also have a role. Studies suggested an association between SARS-CoV-2 and AP due to the virus’s ability to use its glycoproteins to bind to the angiotensin-converting enzyme 2’s (ACE2) receptor, which can be found in the pancreas [2]. According to the World Health Organization (WHO), more than 500 medications have been identified to possibly induce pancreatitis with less than 50 medications determined to have a plausible causality [3]. Paxlovid is a combination of the protease ihibitors (PI) Nirmatrelvir and Ritonavir that has been prescribed in patients diagnosed with COVID-19. Since it’s increased use in the pandemic, more cases of AP have been identified suggesting a link [4].

## Case presentation

**Figure 1:**
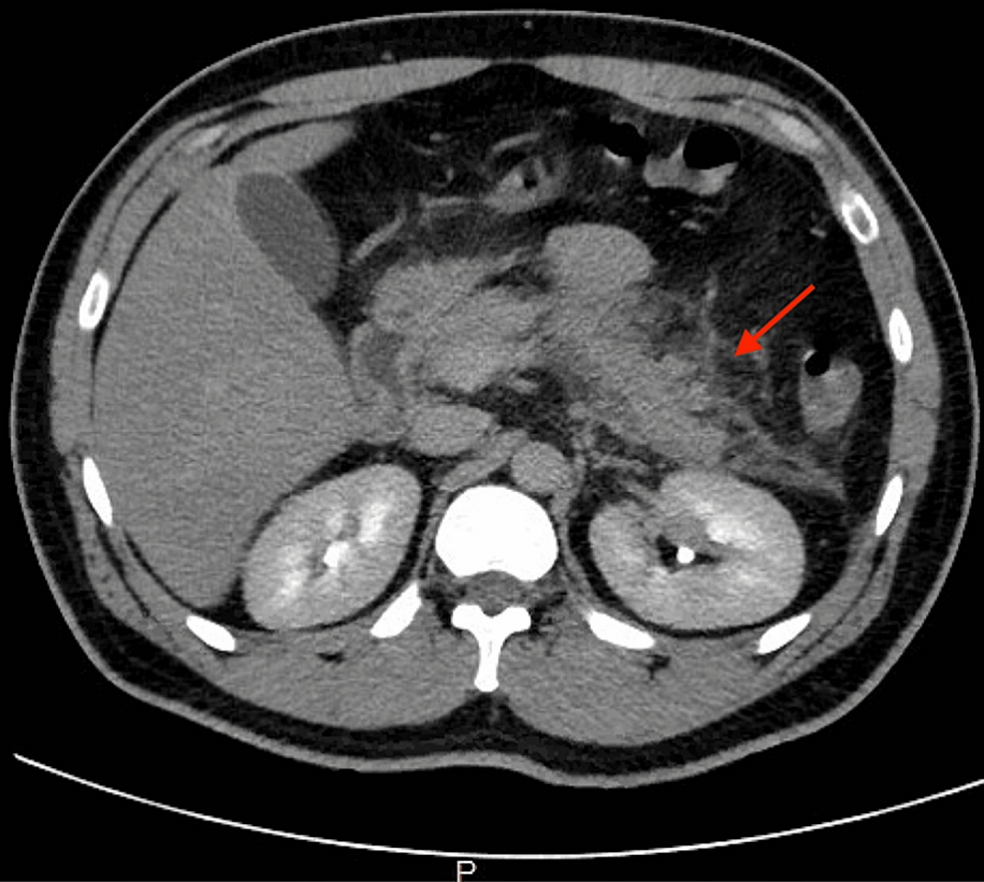
Peri-pancreatic fluid and inflammation predominantly around the tail.

A 37-year-old male with a past medical history of hypertension, hyperlipidemia, and diabetes mellitus type 2 presented with a complaint of sudden-onset, stabbing, continuous epigastric abdominal pain radiating to the back. These symptoms were aggravated by food and activity. He denied any relieving factors but reported associated nausea and fevers up to 102°F. He denied any significant history of alcohol or illicit drug use. Nine days prior to his admission, the patient was diagnosed with COVID-19. He completed a five-day course of Paxlovid (Nirmatrelvir/Ritonavir) with the last dose two days prior to admission. After three days of treatment, his COVID-19 home test was negative. Vitals were normal except for sinus tachycardia. Initial labs are given in Table 1. Ultrasound of the abdomen showed fatty liver and gallbladder sludge. CT of the abdomen with contrast showed edema and fluid around the pancreas greatest around the pancreatic tail concerning AP (Figure 1). Initial management included nil per oral (NPO) status, pain control, and intravenous fluids. During the hospital stay the patient's pain significantly improved and the diet was advanced appropriately. He was eventually discharged home in stable condition with the recommendation of close outpatient follow-up.

**Table 1:** Patient's initial lab results on presentation with normal reference ranges.

Lab Test	Reference Ranges	Result
WBC	3.5 - 10.5 x 10^9^/L	17.30 × 10^9^/L
Hemoglobin	13.5- 17.5 g/dL	12.7 g/dL
Hematocrit	38.8 - 50%	37.7%
Triglycerides	< 150 mg/dl	492 mg/dl
ALT	10 - 50 IU/L	21 IU/L
AST	8 - 48 IU/L	15 IU/L
Total Bilirubin	0.1 - 1.0 mg/dL	0.90 mg/dL
Lipase	10 - 73 U/L	35 U/L
Amylase	60 - 180 U/L	18 U/L
Ethanol	Negative	Negative
COVID-19	Negative	Negative
Urine Drug Screen	Negative	Negative

 

## Discussion

Paxlovid, a combination drug of protease inhibitors Nirmatrelvir and Ritonavir, is an antiviral medication typically given as a five-day course for the treatment of COVID-19. It has a mechanism of action via inhibiting certain proteases in viral replication [5]. It is metabolized by the Cytochrome P450 system and seemingly has significant drug-drug interactions with agents metabolized by the CYP3A4 enzyme [6]. As its use has increased for COVID-19 treatment, there have been increased cases of AP suggesting a possible correlation [4]. Multiple reports in the literature have suggested an association between Ritonavir and AP (Table 2) [3]. A hypothesized theory was that the development of AP was secondary to PI-induced hypertriglyceridemia [7]. COVID-19 infection usually involves the upper and lower respiratory tracts, however, multiple extrapulmonary manifestations have been identified. Increased gastrointestinal system involvement has been noted since the onset of the COVID-19 pandemic, likely due to the abundance of ACE2 in the gastrointestinal tract, creating a nidus for the attachment of the SARS-CoV-2 virus [8]. Liver function test abnormalities with virus detection in stool confirmed the gastrointestinal tract's possible involvement, raising concern for possible oral transmission [8,9]. Pancreatic involvement associated with COVID-19 infection ranges from asymptomatic amylase/lipase elevations to clinically diagnosed AP. Idiopathic AP is the most common cause in patients with COVID-19 infection [2,8]. One study reported the isolation of SARS-CoV-2 RNA from pancreatic pseudocysts: this suggested evidence of viral attachment to the pancreas [10]. One retrospective study on the clinical outcomes of AP in patients with COVID-19 suggested that the association could be due to the abundance of ACE2 receptors in pancreatic beta cells [11]. Pathophysiology seemingly involves direct cytotoxic effects on the pancreas or is secondary to a systemic response. This study concluded that there was a higher incidence of multi-organ failure, morbidity, and mortality in these patients. Hypertriglyceridemia above 500 mg/dL increases the risk of developing the disease and is a common cut-off for initiating medications that would reduce the risk of AP; levels in the 2,000-3,000 mg/dL range are thought to trigger the development of the actual disease [12]. Biliary sludge and gallstones are other potential risk factors [13].

**Table 2:** Table depicting various drugs and drug classes associated with pancreatitis.

Class I	Drug Class
Ritonavir	Protease Inhibitors
Didanosine	Antiviral Agents
Asparaginase	Antineoplastic Agents
Azathioprine	Anticonvulsants
Valproic acid	Anti-Infective Agents
Pentavalent antimonials	Antifungal/Anthelmintic Agents
Pentamidine	Antineoplastic Agents
Mercaptopurine	Gastrointestinal Agents
Mesalamine	Hormonal Agents
Estrogen preparations	Analgesics
Opiates	Antibiotic
Tetracycline	Antineoplastic Agents
Cytarabine	Hormonal Agents
Steroids	Antibiotic
Trimethoprim/Sulfamethoxazole	Anti-infective Agents
Sulfasalazine	Diuretics
Furosemide	Nonsteroidal Anti-inflammatory Drugs
Sulindac	Protease Inhibitors

As seen with our patient, hypertriglyceridemia, biliary sludge, and viral infection were all concurrently present. That, along with the Paxlovid treatment, likely further put him at risk for AP development. However, since TG levels were less than 500 mmol/L and liver enzymes were not elevated, we presume that symptoms were less likely due to these etiologies. Therefore, we hypothesize that Paxlovid initiation could have been the cause of the triggering event leading to AP. This could be further supported by a negative COVID-19 test after three days of treatment with Paxlovid. The timing of AP, two days after completing treatment with Paxlovid, in addition to a negative home test, makes medication-induced AP more likely.

## Conclusions

Drug-induced pancreatitis, difficult to assess, is a notorious complication with many drugs implicated and newer drugs being increasingly identified. Hypertriglyceridemia, biliary sludge, and viral infection with SARS-CoV-2 are risk factors, although may not be very significant in our case. While certain risk factors and triggers do pertain to the cause, drug administration in itself may trigger the acute inflammatory process. The timing of pancreatitis, in our patient, after two days of therapy with repeated negative COVID-19 tests, leads more to the suspicion of Paxlovid-induced pancreatitis. To our knowledge, this is the first case that reported this association. More research is still required to help delineate and confirm associations. This may indicate certain limitations/contra-indications in the future for Paxlovid administration for those already at risk or who have a history of pancreatitis.
